# *Enteromorpha prolifera* Polysaccharides Alleviate Valproic Acid-Induced Neuronal Apoptosis in a Cellular Model of Autism Spectrum Disorder

**DOI:** 10.3390/cimb47100796

**Published:** 2025-09-25

**Authors:** Xulan Zhou, Hui Su, Jiaxuan Chen, Li Liu, Qian Zhou, Xiaochun Xia, Juan Wang

**Affiliations:** 1 Department of Public Health and Medical Technology, Xiamen Medical College, Xiamen 361023, China; zxl@xmmc.edu.cn (X.Z.); gloria20020923@163.com (J.C.); yuzhou202506@163.com (Q.Z.); 2 School of Public Health, Fujian Medical University, Fuzhou 350122, China; suhui625@163.com (H.S.); 990712@fjmu.edu.cn (L.L.)

**Keywords:** *Enteromorpha prolifera* polysaccharide, autism spectrum disorder, network pharmacology, molecular docking, neuronal apoptosis

## Abstract

Autism spectrum disorder (ASD) is a neurodevelopmental condition marked by social/communication deficits and behavioral abnormalities, with neuronal apoptosis and immune-inflammatory dysregulation implicated in its pathogenesis. Marine-derived polysaccharides, particularly those from *Enteromorpha prolifera* (PEPs), exhibit neuroprotective and anti-inflammatory properties—yet their therapeutic potential for ASD remains unexplored. Major monosaccharide components of PEPs were identified as rhamnose, xylose, glucose, glucuronic acid, galactose, and ribose through ion chromatography analysis. Infrared spectroscopy confirmed PEPs as pyranose-type polysaccharides with α-glycosidic bonds and uronic acids, while gel permeation chromatography showed a predominant molecular weight of 3.813 kDa (83.919%). To explore the therapeutic potential of PEPs in ASD, a comprehensive method combining network pharmacology, molecular docking, and in vitro validation was conducted. A total of 235 ASD-related target proteins were predicted, with enrichment analyses indicating significant involvement in pathways such as neuroactive ligand–receptor interaction and the MAPK signaling pathway. In vitro assays using valproic acid (VPA)-induced HT22 neuronal cells showed that PEPs significantly attenuated apoptosis. Western blot analysis further confirmed the downregulation of HSP90AA1, cleaved CASP3/pro-CASP3, p-NF-κB1/NF-κB1, p-AKT1/AKT, and p-mTOR/mTOR, as well as the upregulation of IκBα after PEPs treatment. These findings suggest that PEPs exert neuroprotective effects through the modulation of apoptosis and inflammation-related signaling pathways, supporting their potential as a promising candidate for further study in ASD.

## 1. Introduction

Autism Spectrum Disorder (ASD) is a lifelong neurobiological disorder characterized by difficulties in social interaction, communication impairments, as well as restricted and repetitive patterns of behavior [1]. Clinical symptoms of ASD are highly variable among patients, with significant differences in the severity of social communication impairments and cognitive functions. In 2025, the Autism and Developmental Disabilities Monitoring Network analyzed data from 16 states in the United States and reported that the overall prevalence of ASD in 4–8 year olds increased from 1 in 54 in 2016 to 1 in 31 in 2022 [2]. ASD is a significant economic and psychological burden for society and families. It is a global public health concern because of its increasing prevalence, poor prognosis, high disability rate, and need for long-term rehabilitation. Although there are no specific treatments for ASD, atypical antipsychotic medications can alleviate peripheral symptoms such as aggression and stereotypical behavior [3], or improve anxiety and other mood disorders by targeting serotonin reuptake inhibition [4]. Currently, there are no approved medications to improve the social and cognitive impairments in individuals with ASD [5] or delay the pathological progression of the disease. Current treatment primarily focuses on early behavioral interventions.

Autism development is affected by genetic as well as environmental factors. Advances in high-throughput sequencing technologies in recent years have led to the identification of several candidate ASD risk genes and genetic loci. Mutations in genes encoding synaptic adhesion molecules, including Neurexins and Neuroligins, as well as postsynaptic scaffold proteins such as SHANK family proteins are critical genetic factors involved in ASD [6]. Moreover, genetic differences in neuronal development regulators and signaling pathway factors, such as PTEN, FMR1, and MECP2, are strongly linked to ASD susceptibility [7].

Although genetic factors play a predominant role in ASD, environmental factors also play a crucial role in triggering or exacerbating the development of ASD in genetically susceptible individuals. Maternal prenatal factors are among the most well-studied environmental risk factors in ASD. Maternal Immune Activation (MIA) is a significant risk factor for ASD [8]. Heavy metals in the environment are also significant risk factors for ASD [9]. Furthermore, exposure to certain medications or chemicals during pregnancy can also increase the risk of ASD in the offspring. Exposure to valproic acid (VPA), an antiepileptic drug and mood stabilizer, during pregnancy is significantly associated with an increased risk of ASD in the offspring [10]. Animal models and in vitro cell models exposed to VPA are widely used to investigate the pathogenic mechanisms of ASD. VPA disrupts neural development by altering gene expression, increasing oxidative stress, disrupting synapse formation, influencing the GABAergic system, and altering key signaling pathways such as Wnt/β-catenin [11].

Genetic and environmental risk factors influence the clinical symptoms of ASD by modulating both intracellular and extracellular molecular signaling pathways, thereby affecting various critical stages of brain development. These pathways are involved in the regulation of synaptic function and plasticity [12], neuronal apoptosis [13], gut microbiota dysbiosis [14], neuroimmunity and inflammation [15], oxidative stress [16], and neurotransmitter balance [17].

Natural products such as marine polysaccharides have received significant attention from researchers because of their diverse chemical structures and multi-target activities. *Enteromorpha prolifera* is a plant belonging to the Chlorophyta phylum, Ulvophyceae class, and Ulvaceae family [18]. It is a common green seaweed found along coastlines. Its annual production exceeds 100,000 tons in the coastal areas of Fujian, China. *Enteromorpha prolifera* polysaccharide (PEPs) is a water-soluble sulfated heteropolysaccharide extracted from Enteromorpha. PEPs demonstrates antioxidative, antitumor, hypoglycemic, hypolipidemic, anticoagulant, immunostimulant, antimicrobial, and anti-inflammatory properties [19,20,21,22,23,24]. The oligosaccharides of *Enteromorpha prolifera* exert protective effects on the hippocampal neurons of SAMP8 aging model mice through their antioxidative effects, modulation of neuroprotective protein expression, reduction in neuroinflammation, and regulation of gut microbiota [25]. Few studies have investigated the neuroprotective effects of PEPs. However, there is significant clinical interest in the neuroprotective effects of sulfated polysaccharides from marine algae. Polysaccharides from marine algae demonstrate neuroprotective effects against zinc-induced neuronal damage by inhibiting cellular apoptosis, oxidative damage, and acetylcholinesterase activity [26]. However, despite the extensive research on PEPs in other disease models, their therapeutic potential in neurodevelopmental disorders, particularly in the field of ASD, has not been systematically explored. This study used network pharmacology to identify active ingredients in PEPs with beneficial effects on ASD. Molecular docking technology and in vitro cell-based experiments were used to validate the predictions.

## 2. Materials and Methods

### 2.1. Extraction of Polysaccharides from Enteromorpha prolifera

*Enteromorpha prolifera* powder is produced by Fujian Haixing Health Food Co., Ltd. in Fujian, China, through constant temperature drying at 40 degrees Celsius, followed by crushing and sieving. The yield of *Enteromorpha prolifera* powder is 5% of the starting dry mass. A suspension of *Enteromorpha prolifera* powder, used in aquaculture, was prepared in distilled water at a 1:40 ratio (g:mL; weight-to-volume ratio). The mixture was then heated in a water bath at 80 °C for 4 h. The supernatant was concentrated to 1/3rd of its original volume. The concentrated supernatant was mixed with 95% ethanol in a 1:4 ratio to precipitate PEPs, which was subsequently freeze-dried. The polysaccharide concentration was determined to be 64.9% using the phenol sulfuric acid method. The experiment utilized the same batch of *Enteromorpha prolifera* powder, with the extracted polysaccharides being sealed at 4 degrees Celsius and stored in a dark place.

### 2.2. Analysis of the Monosaccharide Structure of PEPs

A total of 16 monosaccharide standards (fucose, rhamnose, arabinose, galactose, glucose, xylose, mannose, fructose, ribose, galacturonic acid, glucuronic acid, aminogalactose hydrochloride, glucosamine hydrochloride, N-acetyl-D-glucosamine, guluronic acid, and mannuronic acid) were transferred into ampoules. They were mixed with 2 mL of 3 M Trifluoroacetic Acid (TFA), hydrolyzed for 3 h at 120 °C, and dried with a nitrogen blower (Lichen Technology UGC-24M). The powder was mixed with 5 mL of water by vortexing to prepare a standard mother liquor solution.

5 mg of PEPs was weighed and subjected to hydrolysis using an identical method to the monosaccharide standards. The PEPs powder was mixed with 5 mL of water. Then, 50 µL of the PEPs solution was diluted with 950 µL of deionized water and centrifuged at 12,000 rpm for 5 min. The supernatant was analyzed by ion chromatography (ThermoFisher ICS5000, Waltham, MA, USA). Standards were prepared with specific concentrations of different monosaccharide solutions for the estimation of the molar mass of various monosaccharides using the absolute quantitative method. The molar ratio was calculated based on the molar mass of monosaccharides. The chromatographic conditions are shown in Table 1.

### 2.3. Determination of PEPs Molecular Mass

The molecular weight of PEPs was determined using high-performance gel permeation chromatography (HPGPC). HPGPC analysis was carried out in a mobile phase of 0.5 M NaCl at 40 °C, with a flow rate of 0.5 mL/min, using a high-performance liquid chromatography system equipped with a refractive index detector (RID-20A) (Thermo U3000, Waltham, MA, USA) and a tandem gel column (BRT 105-103-101, 8 × 300 mm). Firstly, linear dextran standards were used for analysis under the aforementioned chromatographic conditions. Standard curves for LgMp-RT (peak molecular weight), LgMw-RT (weight-average molecular weight), and LgMn-RT (number-average molecular weight) were plotted. The molecular weight calculation formulas were derived, with the calibration curve equations as follows:

y = −0.5926x + 13.192, R^2^ = 0.9943 for LgMp-RT; y = −0.5995x + 13.286, R^2^ = 0.9944 for LgMw-RT; and y = −0.5965x + 13.237, R^2^ = 0.9933 for LgMn-RT. Subsequently, dissolve 5 mg of PEPs in 1 mL of the mobile phase, followed by sonication for 10 min, centrifugation at 12,000 rpm for 10 min, and filtration of the supernatant through a 0.22 μm filter membrane. By substituting the retention times of PEPs into the formula, molecular weights (Mp, Mw, Mw) can be obtained. The detection results are plotted with retention time on the x-axis and differential signal on the y-axis.

### 2.4. The Infrared Characteristic Spectrum of PEPs

Weighing 2 mg of PEPs and 200 mg of KBr meticulously, the samples were thoroughly ground and pressed into pellets. Subsequently, Fourier-transform infrared spectroscopy (FT-IR650, Tianjin Port East Technology Development Co., Ltd., Tianjin, China) was employed to scan and record spectra within the wavenumber range of 4000 cm^−1^ to 500 cm^−1^.

### 2.5. Network Pharmacology Analysis

The SMILES for each monosaccharide in PEPs were retrieved from the PubChem database (https://pubchem.ncbi.nlm.nih.gov/, accessed on 17 March 2025) and used to predict the targets of the monosaccharides in PEPs from the following four databases: SwissTarget Prediction (http://swisstargetprediction.ch/, accessed on 18 March 2025), PharmMapper (https://lilab-ecust.cn/pharmmapper, accessed on 12 May 2025), SuperPred (https://prediction.charite.de/, accessed on 1 April 2025), and Similarity Ensemble Approach (https://sea.bkslab.org/, accessed on 3 April 2025). The target names were standardized using the UniProt database (https://www.uniprot.org/, accessed on 3 April 2025) and duplicate entries were eliminated to determine the action targets of PEPs. The search term “autism” was used to find targets of ASD in the GeneCards (https://www.genecards.org/, accessed on 5 April 2025), OMIM (https://www.omim.org/, accessed on 5 April 2025), and TTD (https://db.idrblab.net/ttd/, accessed on 5 April 2025) databases. The target names were standardized using the UniProt database and duplicate entries were eliminated to obtain the targets of ASD. Then, VennDiagram in R language (R-4.4.3, RStudio 2024.12.1) was used to generate the intersection target map of the PEPs and ASD targets to identify the ASD-related PEPs targets.

### 2.6. Protein–Protein Interaction (PPI) Network Analysis

The ASD-related PEPs targets were imported into the STRING database (https://cn.string-db.org/, accessed on 16 May 2025) and a PPI network was generated with “Homo sapiens” as the species type and a minimum required interaction score ≥ 0.4 as the threshold parameter. The free targets were hidden or concealed. The data was exported in the TSV format. Subsequently, this file was imported into the Cytoscape 3.9.1 software, and a PPI network diagram of the overlapping ASD-related PEPs targets was generated. To assess the importance of the ASD-related PEPs targets, the Network Analyzer plugin was used to determine the degree, betweenness centrality values, closeness centrality values, and neighborhood connectivity values. Then, the CytoHubba plugin and the Maximum Clique Centrality (MCC) algorithm were used to identify the top 10 hub genes.

### 2.7. Gene Ontology (GO) and Kyoto Encyclopedia of Genes and Genomes (KEGG) Pathway Enrichment Analysis

GO functional enrichment and KEGG pathway enrichment analysis of the intersecting PEPs–ASD targets was performed in the DAVID platform (https://davidbioinformatics.nih.gov/, accessed on 16 May 2025) to identify the related transcription factors and regulatory genes of the ASD-related PEPs targets. The results were visualized and analyzed using R language.

### 2.8. MCODE Analysis

The MCODE algorithm was used to analyze the PPI network and identify gene clusters with similar functions. The key targets were uploaded to the Metascape platform (https://metascape.org/gp/index.html, accessed on 19 May 2025) and assessed with *p*-value < 0.01, at least 3 counts, and enrichment factor > 1.5 as cutoff criteria. Enriched terms were clustered based on their similarity in membership. This enabled the identification of highly connected subnetworks in the PPI network.

### 2.9. Molecular Docking

The primary active components of PEPs and the central targets were chosen based on the overlapping targets for molecular docking. Firstly, the SDF structure file of the core components was obtained from the PubChem database and converted to a PDB format file using Open Babel 2.3.2. The target proteins were then pretreated. The 3D structure file of the protein target in PDB format was retrieved from the PDB database (https://www.rcsb.org/, accessed on 6 May 2025). Subsequently, the protein target underwent processing, including desolvation, hydrogenation, and setting of appropriate active pockets and docking parameters using the AutoDockTools 1.5.7 software. Molecular docking simulations were performed using the “AutoDock Vina” module. The docking method employed was semi-flexible docking with an exhaustiveness setting of 20. PyMOL 3.1.0 software was used for visualization and analysis.

### 2.10. Cell Models and Drug Interventions

#### 2.10.1. Cell Culture

HT22 cells were purchased from the Shanghai Cell Bank (Shanghai, China) and were cultured in DMEM complete medium containing 10% fetal bovine serum and 1% penicillin–streptomycin at 37 °C and 5% CO_2_ in a humidified incubator. Experiments were conducted with cells in a logarithmic growth phase with a confluency of over 80%.

#### 2.10.2. CCK-8 Assay

The toxic effect of PEPs on the growth of HT22 cells was assessed using the CCK8 assay. Briefly, 5 × 10^3^ cells per well were seeded in a 96-well plate and incubated for 24 h. Then, different concentrations of PEPs (0, 25, 50, 75, 100 μg/mL) were added and the cells were incubated for a further 48 h. Then, 10% CCK8 solution (Apexbio, K1018, Houston, TX, USA) was added, and the cells were further incubated for 1 h to develop color. Subsequently, optical density (OD) was measured at 450 nm using a microplate reader to determine the cytotoxic concentrations of PEPs. Furthermore, HT22 cells were treated with 2 mM VPA (Sigma, P4543, Saint Louis, MO, USA) for 24 h and used as the model group. Different concentrations of PEPs (0, 25, 50, 75, 100 μg/mL) were added concurrently with VPA intervention. Each group contained 6 replicate wells. After 48 h of incubation, 10% CCK8 solution was added. Then, after incubation for color development, the optical density (OD) was measured at 450 nm using a microplate reader. All experiments were repeated thrice for statistical analysis.

#### 2.10.3. Effects of PEPs on VPA-Induced Neuronal Apoptosis

Cellular apoptosis was assessed using the Cell Apoptosis Detection Kit (Abbine, KTA0002, Wuhan, China). Cells were seeded at a density of 1 × 10^5^ cells per well in a 6-well plate. The experimental groups included a control group, VPA group, and PEPs group. The control group was grown in complete culture medium, whereas the VPA group and PEPs group were grown in complete medium containing 2 mmol/L VPA and four different doses of PEPs (25, 50, 75, 100 μg/mL), respectively. After 48 h of incubation, the cells were collected by trypsinization without EDTA, washed twice with ice-cold PBS, resuspended in 500 µL 1× Binding buffer, and stained with 5 µL Annexin V-AbFluor™ 488 and 2 µL propidium iodide (PI). The cell suspension was then gently mixed and incubated in the dark at room temperature for 15 min. The percentage of apoptosis was evaluated by flow cytometry. Quadrant analysis distinguished necrotic cells (Q1), late-stage apoptotic cells (Q2), early-stage apoptotic cells (Q3), and normal cells (Q4). Gating was established using the following groups: the VPA group labeled with PI (PI positive control), the VPA group labeled with FlTC Annexin V (Annexin V positive control), and the unstained group (negative control). We performed flow cytometry analysis within 30 min, using AbFluor™ 488 with an excitation wavelength of 491 nm/emission wavelength of 517 nm and PI with an excitation wavelength of 535 nm/emission wavelength of 617 nm. In order to guarantee the reliability and reproducibility of the results, all experiments were carried out in triplicate.

#### 2.10.4. Western Blotting

The total protein was extracted from the different groups of cells. Briefly, cells were manually homogenized using the RIPA buffer (Cell Signaling Technology, Danvers, MA, USA), in the presence of protease inhibitor (PMSF). Protein concentration was estimated using the BCA method. Equal amounts of total protein lysates were separated on an 8–12% SDS-PAGE gel and transferred onto a polyvinylidene fluoride (PVDF) membrane. The membrane was blocked with 5% skim milk at room temperature for 1 h. Then, the blot was probed overnight at 4 °C with primary antibodies against caspase-3 (1:1000, Cell Signaling Technology, Danvers, MA, USA), cleaved caspase-3 (1:1000, Cell Signaling Technology, Danvers, MA, USA), HSP90AA1 (1:1500, UpingBio, Hangzhou, China), AKT1 (1:2000, Cell Signaling Technology, Danvers, MA, USA), p-AKT-S473 (1:2000, Abcam, Cambridge, UK), p-AKT-T308 (1:1000, ABclonal, Wuhan, China), lκBα (1:1000, Cell Signaling Technology, Danvers, MA, USA), NF-κB1 (1:1000, UpingBio, Hangzhou, China), p-NF-κB1 (1:1500, UpingBio, Hangzhou, China), mTOR (1:3000, Cell Signaling Technology, Danvers, MA, USA), p-mTOR (1:3000, Cell Signaling Technology, Danvers, MA, USA), GAPDH (1:10,000, Proteintech, Rosemont, IL, USA), or β-actin (1:10,000, Abbkine, Wuhan, China). Then, peroxidase-conjugated goat anti-rabbit or anti-mouse immunoglobulin G secondary antibody (1:10,000, Abcam, Cambridge, UK) was used at room temperature for 1 h. After being exposed to an ECL reagent, the bands were visualized and imaged with the ECL imaging system from Bio-Rad in Berkeley, CA, USA.

### 2.11. Statistical Analysis

Statistical data analysis was performed using the IBM SPSS 27.0 and GraphPad Prism 9.0 software. The data was presented as mean ± standard deviation (S.D.). One-way ANOVA was used to compare the data between multiple groups. Multiple range testing by Tukey’s method was conducted for comparing means. Statistical significance was set at *p* < 0.05. The * and # notations denote comparisons with the control and model groups, respectively.

## 3. Results

### 3.1. Monosaccharide Composition of PEPs

**Figure 1:**
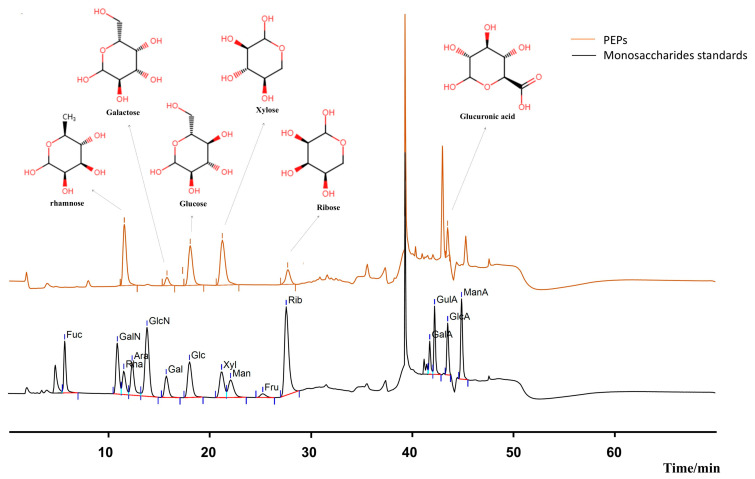
The monosaccharide composition of PEPs. (N-Acetyl-D-glucosamine will decompose under hydrolysis conditions at 120 °C, so it cannot be presented in the mixed standard chromatogram).

Based on the ion chromatography results, six polysaccharides were identified in PEPs, namely, rhamnose, xylose, glucose, glucuronic acid, galactose, and ribose with a molar ratio of 3.88:3.2:1.46:0.58:0.49:0.56 (Figure 1).

### 3.2. Molecular Weight of PEPs

**Figure 2:**
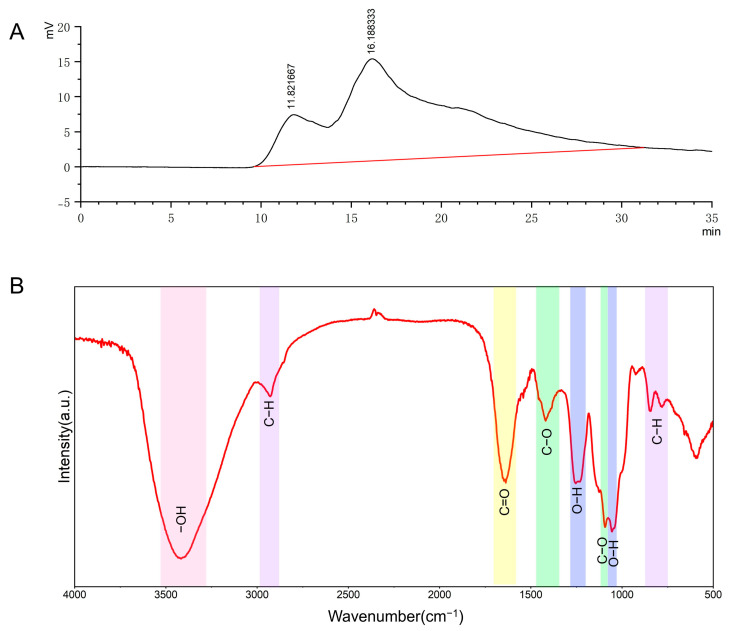
Structural analysis of PEPs. (**A**) Molecular weight, (**B**) FTIR.

Polysaccharides, as conventional polymers, consist of a mixture of homologues with varying molecular weights. Therefore, the molecular weight of polysaccharides is commonly characterized by number-average molecular weight (Mn), weight-average molecular weight (Mw), z-average molecular weight (Mz), peak molecular weight (Mp), and polydispersity index (PDI). After calculating the retention time of the samples using the formula, the results indicate that the PEPs are heterogeneous polysaccharides, consisting of two components. One component with a molecular weight (Mw) of 3.813 kDa accounts for 83.919% of the total, making it the main component of the PEPs and a low molecular weight polysaccharide. The other component, with a molecular weight (Mw) of 1.58 × 10^3^ kDa, represents 16.081% and is a high molecular weight polysaccharide (Figure 2A, Table 2).

### 3.3. Analysis of PEPs Using Infrared Spectroscopy

The IR spectrum of PEPs is shown in Figure 2B, with a broad and intense absorption peak appearing at 3200–3600 cm^−1^, indicative of the stretching vibration absorption peak of -OH. The presence of characteristic peaks suggests that the sample under investigation is a polysaccharide substance. The absorption peak at 3417 cm^−1^ is attributed to the stretching vibration of O-H, characteristic of sugars. The absorption peak at 2927 cm^−1^ corresponds to the stretching vibration of C-H bonds, characteristic of carbohydrates. An absorption peak at 1637 cm^−1^, possibly assigned to the C=O stretching vibration, suggests the presence of aldonic acid in the polysaccharide. Absorption peaks are observed at 1417 cm^−1^ and 1093 cm^−1^, potentially associated with C-O stretching vibrations, particularly the band at 1000–1150 cm^−1^, likely attributed to C-O-C glycosidic bond vibrations. Absorption peaks at 1253 cm^−1^, 1232 cm^−1^, and 1054 cm^−1^ suggest the presence of O-H bending vibrations or C-O-C bond deformations in the PEPs, indicating the presence of furanose sugar rings. The absorption peak at 848 cm^−1^ may be attributed to the C-H bending vibration of the α-end group, indicating that the glycosidic linkage configuration of PEPs is predominantly in the α-configuration. Infrared spectroscopy analysis indicates that PEPs are pyranose polysaccharides containing α-glycosidic bonds and uronic acids.

### 3.4. Analysis of Potential ASD-Related PEPs Targets

#### 3.4.1. Screening the Main Active Components of PEPs

**Figure 3:**
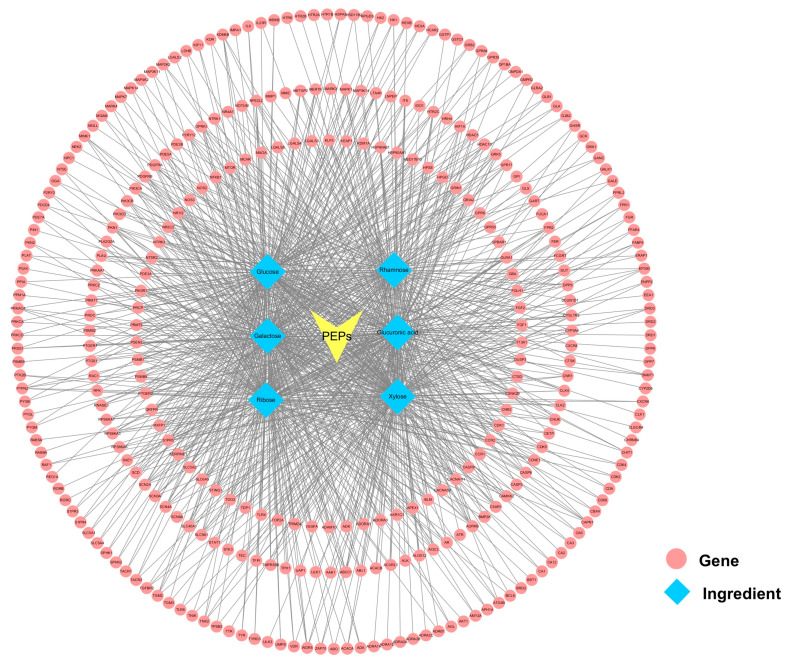
Components-targets-network of PEPs.

**Figure 4:**
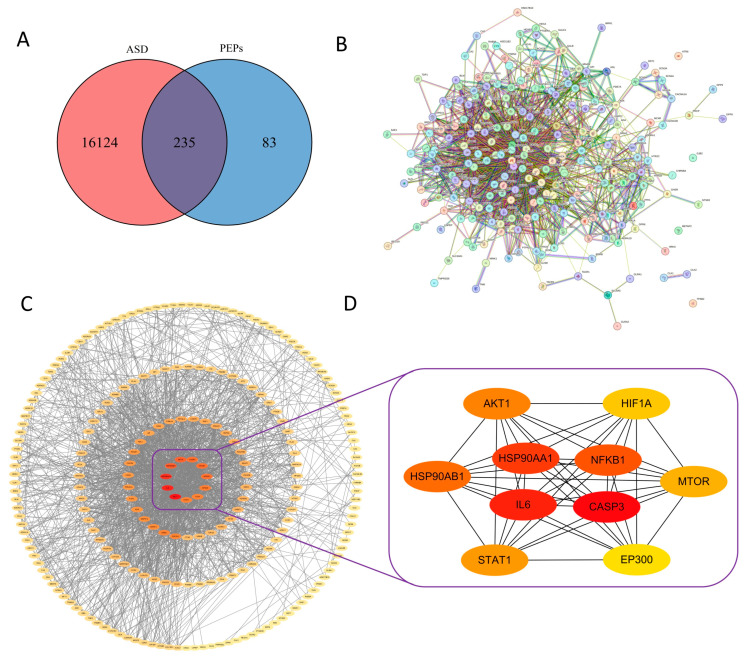
PPI network analysis of ASD-related PEPs targets. (**A**) Venn diagram shows identification of ASD-related PEPs targets. (**B**) PPI network of potential ASD-related PEPs targets. (**C**) Interaction network of ASD-related PEPs target proteins. Darker color indicates more critical targets. (**D**) Core target PPI diagram. The core genes were screened based on the Maximum Clique Centrality algorithm. Darker color indicates more critical targets.

33 PEPs targets were identified from the SwissTarget Prediction database, 232 PEPs targets from the SuperPred database, 40 PEPs targets from the PharmMapper database, and 37 PEPs targets from the Similarity Ensemble Approach (SEA) using the monosaccharides of PEPs as active ingredients. As shown in Figure 3, the six monosaccharide components of PEPs have similar target numbers, with xylose being the most abundant with 178 targets. After eliminating duplicate targets, 318 PEPs targets were identified. Furthermore, 16,349 ASD-related targets were identified from the GeneCards database, 24 ASD-related targets from the OMIM database, and 12 ASD-related targets from the TTD database. After removing duplicate targets, 16,359 ASD-related targets were identified. By intersecting the 318 PEPs targets and 16,359 ASD-related targets, 235 intersecting targets were identified (ASD-related PEPs targets) by VennDiagram in R language (Figure 4A).

#### 3.4.2. PPI Network Analysis Results

Using a medium confidence threshold of ≥0.400, a PPI network was generated with 233 nodes and 1761 edges (Figure 4B,C). The proteins OPRK1 and LDHB were not included in the STRING database. Subsequently, the interacting targets were imported into Cytoscape. The Maximum Clique Centrality (MCC) algorithm in the “cytoHubba” plugin was then used to identify the top ten core genes. Proteins with higher degree values in the PPI networks at the nodes were represented with darker colors. The top ten core ASD-related PEPs target proteins were Cysteine protease 3 (CASP3), Interleukin-6 (IL6), heat shock protein 90 kDa alpha (cytosolic) class A member 1 (1HSP90AA1), nuclear factor kappa B subunit 1 (NF-κB1), Recombinant Heat Shock Protein 90 kDa Alpha B1 (HSP90AB1), AKT serine/threonine kinase 1 (AKT1), signal transducer and activator of transcription 1 (STAT1), Mammalian Target of Rapamycin (mTOR), hypoxia-inducible factor 1-alpha (HIF1A), and E1A binding protein p300 (EP300) (Figure 4D, Table 3).

### 3.5. Functional Enrichment Analysis of ASD-Related PEPs Targets

**Figure 5:**
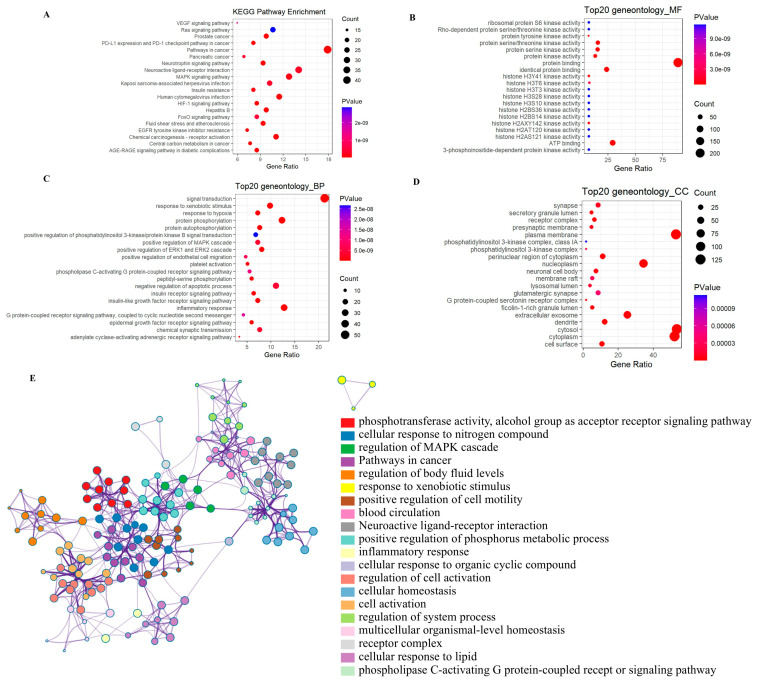
KEGG and GO functional enrichment analysis of ASD-related PEPs target genes. (**A**) KEGG pathway enrichment analysis shows the potential pathways targeted by PEPs related to ASD. (**B**) GO enrichment analysis shows the molecular functions (MF) enriched by the ASD-related PEPs target genes. (**C**) GO enrichment analysis of biological processes (BP) enriched by the ASD-related PEPs target genes. (**D**) GO enrichment analysis of cellular components (CC) enriched by the ASD-related PEPs target genes. (**E**) MCODE analysis of core target genes for PEPs. Enrichment results are displayed by the network plot. The node size indicates the degree of enrichment.

Functional pathway enrichment analysis was performed using the David platform (Figure 5A) and showed that the ASD-related PEPs targets were enriched in pathways such as pathways in cancer, neuroactive ligand–receptor interaction, and the MAPK signaling pathway. GO functional enrichment analysis of the ASD-related PEPs targets was performed to identify the top 20 biological processes (BP), molecular functions (MF), and cellular components (CC). ASD-related PEPs targets were enriched in BP such as signal transduction, inflammatory response, protein phosphorylation, and negative regulation of apoptotic process (Figure 5B), as well as MF such as protein binding, ATP binding, and identical protein binding (Figure 5C), and CC such as cytosol, plasma membrane, cytoplasm, and nucleoplasm (Figure 5D).

### 3.6. MCODE Analysis of ASD-Related PEPs Targets

The mechanisms by which PEPs regulates ASD were further investigated through construction of a modular network of 235 ASD-related PEPs interacting genes using the MCODE algorithm in Metascape, in order to identify core treatment targets. MCODE analysis demonstrated that the core treatment targets of PEPs were phosphotransferase activity, cellular responses to nitrogen compounds, regulation of MAPK cascades, pathways in cancer, cellular responses to lipids, neuroactive ligand–receptor interaction, and inflammatory responses (Figure 5E).

### 3.7. Molecular Docking Analysis Results

**Figure 6:**
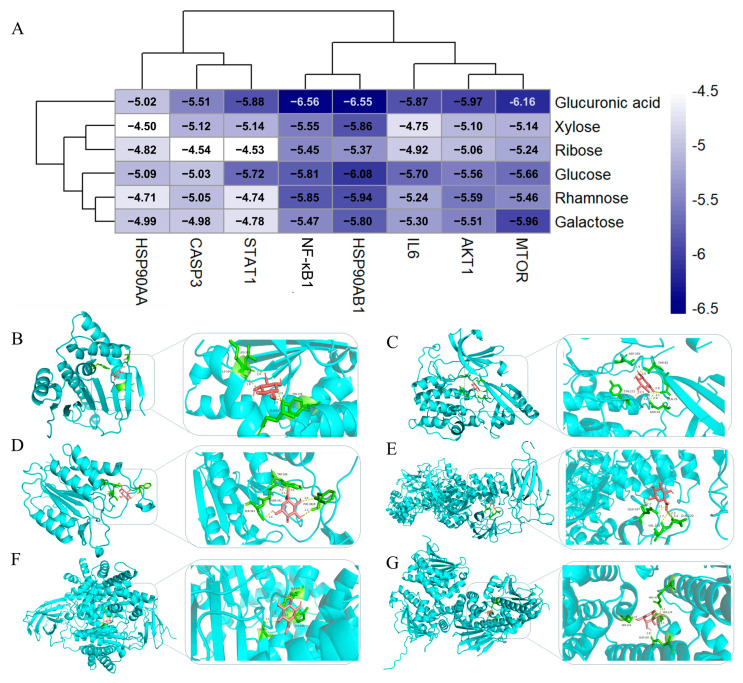
Molecular docking results. (**A**) Molecular docking energy of PEPs target proteins. (**B**) Visualization of docking results between glucose and HSP90AA1. (**C**) Visualization of docking results between glucuronic acid and AKT1. (**D**) Visualization of docking results between glucuronic acid and CASP3. (**E**) Visualization of docking results between glucuronic acid and NF-κB1. (**F**) Visualization of docking results between glucuronic acid and mTOR. (**G**) Visualization of docking results between rhamnose and HSP90AB1.

To investigate the interaction between key target proteins and PEPs compounds, all the monosaccharide components of PEPs were selected as ligands, namely rhamnose, xylose, glucose, glucuronic acid, ribose, and galactose. The selection criteria are as follows: the organism is Homo sapiens, and the structure determination method is X-Ray Diffraction. Structures that were prioritized for downloading in PDB format met the following criteria: resolution was less than 3 Å (1 Å = 0.1 nm) [27,28]. To validate the predicted key targets, LibDock molecular docking was performed on the selected core targets using Discovery Studio 2019 software. The 3D protein-ligand complex structures of core targets were retrieved from the PDB database. The native ligand was removed from the protein 3D structure, the protein and ligand structures were processed separately by removing all water molecules, force field optimization was performed after handling the protein structure and hydrogenation, the binding site was determined, and redocking was performed to validate molecular docking of the core targets. Through the above method, we identified the following protein structures: CASP3 (PDB ID: 1RE1, RMSD = 0.9514 Å), HSP90AA1 (PDB ID: 3B25, RMSD = 0.8184 Å), AKT1 (PDB ID: 4ejn, RMSD = 1.4494 Å), HSP90AB1 (PDB ID: 7ULJ, RMSD = 1.3184 Å), and mTOR (PDB ID: 4HVB, RMSD = 1.5524 Å), all with root mean square deviation (RMSD) values less than 2.0 Å, indicating the reliability of this method. In addition, we have identified the docking structure of three other proteins through the literature: IL6 (PDB ID: 1alu) [29], NF-κB 1 (PDB ID: 1nfi) [30], and STAT1 (PDB ID: 7nuf) [31]. For detailed parameters of the docking box, refer to Supplementary Table S1. The docking results are shown in Figure 6A. Hydrogen bonding between various active components of PEPs and protein targets were evaluated. Glucuronic acid demonstrated the strongest binding with NF-κB 1 and a binding energy of −6.56 kcal/mol (Figure 5E). Glucuronic acid interacted hydrophobically with specific amino acids (GLN-247, VAL-219, and GLN-220) in NF-κB1, thereby suggesting its potential binding with the key protein targets of ASD, as illustrated in Figure 6E. Furthermore, the majority of monosaccharides exhibited affinity for these proteins, with binding energies of ≤−5 kcal/mol (Figure 6A). Molecular docking diagrams depicting interactions between various monosaccharides and proteins are illustrated (Figure 6B–G). This further validated the accuracy of network pharmacology screening.

### 3.8. PEPs Suppresses VPA-Induced Cytotoxicity in HT22 Cells

**Figure 7:**
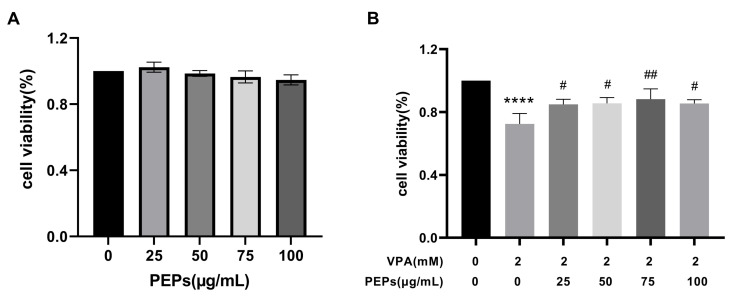
Cytotoxicity and anti-VPA effects of PEPs on the HT22 cells (*n* = 3/each group). (**A**) Effects of different concentrations of PEPs on the viability of HT22 cells. (**B**) Effects of different concentrations of PEPs on the viability of HT22 cells treated with 2 mM VPA compared with the model group (VPA-treatment group). **** *p* < 0.0001 vs. the control; # *p* < 0.05, ## *p* < 0.01 vs. model group (VPA treatment group).

Exposure of HT22 cells to different concentrations of PEPs (0, 25, 50, 75, 100 μg/mL) did not result in cytotoxic effects (Figure 7A). Compared to the model group, various doses of PEPs intervention can ameliorate VPA-induced HT22 cell toxicity (*p* < 0.0001, η^2^ = 0.834). Improvement in cytotoxicity was significantly observed with doses ranging from 25 to 100 μg/mL PEPs, with the most notable enhancement seen at a concentration of 75 μg/mL PEPs. (Figure 7B).

### 3.9. PEPs Suppresses VPA-Induced Apoptosis in the HT22 Cells

**Figure 8:**
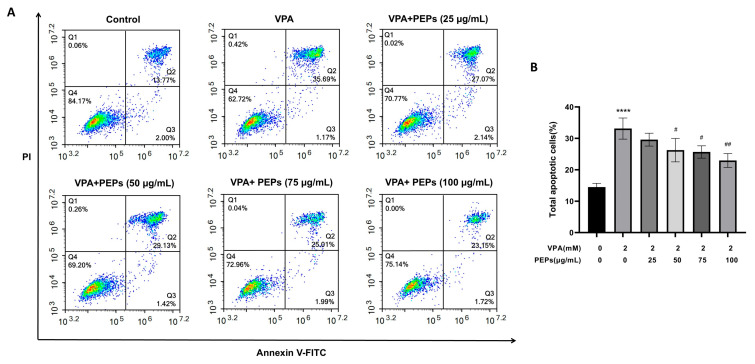
Effects of PEPs on VPA-induced apoptosis in HT22 cells (*n* = 3/each group). (**A**) Flow cytometry analysis results show the effects of apoptosis in the VPA-treated HT22 cells. (**B**) Statistical analysis of data on HT22 cell apoptosis induced by VPA in PEPs. **** *p* < 0.0001 vs. the control, # *p* < 0.05, ## *p* < 0.01 vs. the VPA.

Flow cytometry analysis showed that VPA significantly increased apoptosis of HT22 cells (*p* < 0.0001, η^2^ = 0.885). Compared with the VPA group, PEPs reduced the apoptotic rate of VPA-treated HT22 cells in a concentration-dependent manner; higher concentrations of PEPs (100 μg/mL) were more effective in decreasing apoptosis of VPA-treated HT22 cells (Figure 8A,B).

### 3.10. Effect of PEPs on the Modulation of Critical Target Proteins in HT22 Cells

**Figure 9:**
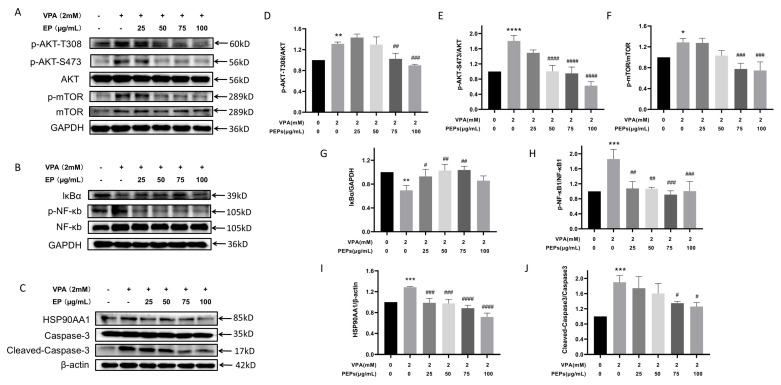
Western blot analysis shows the effects of different concentrations of PEPs on the expression levels of key target proteins in HT22 cells (*n* = 3/each group). (**A**) The Western blot image of p-AKT-T308, p-AKT-S473, AKT, p-mTOR, and mTOR proteins. (**B**) The Western blot image of lκBα, p-NF-κB, and NF-κB proteins. (**C**) The Western blot image of the expression levels of HSP90AA1, Caspase3, and Cleaved-Caspase3. (**D**–**J**) Protein expression levels of p-AKT-T308/AKT, p-AKT-S473/AKT, p-mTOR/mTOR, lκBα, HSP90AA1, and Cleaved-Caspase3/Caspase3. * *p* < 0.05, ** *p* < 0.01, *** *p* < 0.001, **** *p* < 0.0001 vs. the control, # *p* < 0.05, ## *p* < 0.01, ### *p* < 0.001, #### *p* < 0.0001 vs. the VPA.

Western blotting analysis was performed to validate the network pharmacology and molecular docking results by assessing the levels and activities of key target proteins such as HSP90AA1, CASP3, NF-κB1, AKT1, and mTOR. Western blot analysis results (Figure 9A,B) demonstrated that the expression levels of p-AKT-T308/AKT, p-AKT-S473/AKT, p-mTOR/Mtor, p-NF-κB1/NF-κB1, HSP90AA1, and cleaved-CASP3/pro-CASP3 were significantly higher in the model group compared to the control group. However, PEPs intervention significantly reduced the expression levels of these proteins, with more pronounced effects observed for the 50–100 μg/mL doses of PEPs. The detection of lκBα was carried out due to its association with classical NF-κB activation. The findings indicated a significant decrease in lκBα expression with VPA treatment, whereas PEPs (25–75 μg/mL) led to a significant increase in lκBα expression.

## 4. Discussion

ASD is a complex neurodevelopmental disorder that involves interplay between genetic, environmental, and neurobiological factors. Effective treatment options are lacking for patients with ASD, because the underlying mechanisms are not well understood. Therefore, current drug therapies have limited efficacy and are often accompanied by side effects [32]. In recent years, the development of novel treatment strategies derived from natural products has garnered widespread attention. Both *Echinacea purpurea* hydroalcoholic extract and *Ocimum basilicum* L. extract exhibit anti-neuroinflammatory and antioxidant effects to reverse social behavior and repetitive stereotyped actions in autism-like mice [33,34]. Resveratrol, a polyphenolic bioactive compound, alleviates mitochondrial dysfunction and inflammatory cytokine release in an autism rat models, thereby ameliorating neurobehavioral and biochemical deficits associated with autism spectrum disorders [35]. Studies on natural products for ASD are gaining momentum.

*Enteromorpha prolifera*, a seaweed consumed as food and traditional medicine, is rich in nutrients and has shown great potential in the development of novel medical treatments. However, *Enteromorpha prolifera* blooms can negatively impact the growth of other marine organisms. Decomposition of *Enteromorpha prolifera* depletes oxygen in seawater and is a significant threat to shellfish cultivation in intertidal zones. Therefore, rational utilization of *Enteromorpha prolifera* can improve the environment and benefit humanity. Since PEPs demonstrates broad biological activity, this study integrated network pharmacology prediction, molecular docking, and in vitro cellular experiments to investigate the molecular mechanisms by which PEPs mitigates ASD pathology. Our research findings suggest that PEPs exerts its beneficial effects through multiple targets and pathways, which inhibit VPA-induced neuronal apoptosis.

Ion chromatography analysis demonstrated that the main components of PEPs polysaccharides were primarily rhamnose, followed by xylose, and significant amounts of glucose, glucuronic acid, galactose, and ribose. Research has shown that the physical and chemical properties of polysaccharides, such as the composition of monosaccharides, are factors that determine their varying biological activities [36]. The carboxyl group of aldonic acids (such as glucuronic acid) can provide hydrogen atoms, possess electron-donating ability, and chelate transition metal ions to promote oxidation reactions, thereby giving polysaccharides strong free radical scavenging ability and antioxidant activity [37]. Polysaccharides rich in mannose and glucose typically exhibit strong immune activity [38]. The anti-inflammatory activity of rhamnose is related to polysaccharides [39]. The variations in size, configuration, charge, and reactivity among different monosaccharides constitute the origins of the diversity in polysaccharide structures. Although screening using monosaccharides as active targets overlooks the influence of polysaccharide advanced structures on their biological activity, it can still provide some insights into the potential mechanisms of improvement in diseases, and therefore is widely accepted and used by most scholars [40,41,42,43].

Molecular weight is another important parameter that affects the activity of polysaccharides. In this study, the main peak molecular weight of PEPs was measured to be 3.813 kDa, which belongs to the category of low molecular weight polysaccharides. Compared to PEPs reported in other studies (ranging in molecular weight from tens to over a thousand kDa) [44,45], the PEPs obtained in this study have lower molecular weights. Such discrepancies could arise from varying sources such as harvesting times, growth conditions, or extraction methods. It is generally believed that low molecular weight polysaccharides have better water solubility and bioavailability, making it easier to penetrate biological barriers and exhibit stronger biological activity [46]. This could be partly due to PEPs having a strong anti-apoptotic effect on nerve cells. Research has shown that PEPs contains polysaccharides with α-glycosidic bonds in their furanose rings, and the glycosidic bonds play a crucial role in their biological activity [47]. PEPs also contains uronic acid, making it an acidic polysaccharide, and the presence of acidic groups typically enhances the solubility and biological activity of polysaccharides [48].

Network pharmacology analysis results identified multiple key ASD-related PEPs targets, including HSP90AA1, IL6, CASP3, NF-κB1, AKT1, and mTOR. The binding strength between monosaccharides and these proteins was somewhat confirmed through molecular docking, but this evaluation should be considered just one reference point, as the biological activity of polysaccharides is not solely determined by monosaccharides. These targets are part of a complex signaling network that regulates core biological processes such as cell survival, apoptosis, and inflammation. Subsequently, in vitro experiments validated these predictions.

This study reveals that PEPs significantly improves VPA-induced apoptosis in HT22 neuronal cells. Neuronal apoptosis is a physiological process during the development of the nervous system and plays a critical role in regulating the number of neurons by eliminating incorrectly connected neurons and shapes precise neural circuits. About half of the neurons generated during brain development are eliminated by apoptosis to ensure the correct structure and function of the nervous system. However, abnormal activation or dysregulation of this process can result in excessive neuronal loss, thereby disrupting the normal development of brain structure and function and cause cognitive or social behavioral abnormalities in children with ASD [49]. Neuronal apoptosis plays a crucial role in the onset and progression of ASD. In-depth analysis of brain tissues from autistic children in the National Institute of Child Health and Human Development brain bank demonstrated elevated levels of cleaved caspase-8 and PARP as well as significant neuronal apoptosis in the hippocampus, cerebellum, and frontal cortex regions [13]. Numerous ASD animal models have also confirmed that the expression levels of apoptosis biomarkers are elevated in the brain neurons [50]. Apoptosis is associated with neuroinflammation, oxidative stress, and cell cycle regulation. Therefore, dysregulation of apoptosis plays a significant role in the aberrant neurodevelopment underlying ASD [51]. In this study, PEPs significantly inhibits neuronal cell apoptosis in the VPA-induced neuronal injury model. PEPs treatment significantly reduces the cleaved-CASP3-to-pro-CASP3 ratio, indicating its ability to inhibit caspase-3 activation and block the execution phase of apoptosis. In previous reports, it was observed that in animal models, intervention with VPA resulted in an increase in TUNEL-positive cells in the hippocampal region, prefrontal cortex, cerebellum, and neocortex [50,52]. Additionally, changes in apoptosis-related proteins were detected in these areas, such as significantly elevated levels of activated Caspase-3 and an increased Bax/Bcl-2 ratio [53,54]. These research findings are consistent with what we observed at the cellular level, indicating that PEPs can protect nerve cells and alleviate excessive neuronal apoptosis. Therefore, PEPs are a promising candidate for further study in ASD.

This study demonstrates that PEPs inhibits phosphorylation of AKT and mTOR. This suggests that PEPs restores cellular homeostasis by modulating this crucial cell survival pathway. The AKT/mTOR signaling pathway is a key regulator of cellular growth, proliferation, and survival. Its aberrant activation participates in the pathology underlying several neurodevelopmental disorders, including ASD. During brain development, precise regulation of this pathway is critical crucial for key neural developmental processes such as neurogenesis, neuronal migration, axon guidance, dendrite formation, and synaptic plasticity [55]. Abnormal activation of the mTOR signaling pathway is closely associated with increased excitatory synaptic density and functional hyperconnectivity of the cortico-striatal circuit in the brains of individuals with ASD, but pharmacological inhibition of mTOR mitigates these deficits [56]. Contactin-associated protein-like 2 (CNTNAP2) is one of the first and most widely replicated genes associated with autism susceptibility [57]. Cntnap2-deficient mice exhibit core symptoms of ASD, including social difficulties and repetitive behaviors, which are associated with aberrant activation of the AKT/mTOR signaling pathway. Furthermore, inhibition of the AKT/mTOR signaling pathway ameliorates the core symptoms of ASD [58]. In preclinical studies, the mTOR inhibitor rapamycin reverses behavioral and molecular deficits in various ASD animal models [59]. Furthermore, natural products and medications such as Spirulina platensis [60], Taurine [61], and Anethole [62] ameliorate disease symptoms in ASD models. Computer simulations have been used to screen compounds that can target key proteins in the AKT/mTOR signaling pathways, such as AKT1 [63]. Several studies have suggested that precise modulation of the AKT/mTOR pathway activity may offer a novel, molecularly based targeted therapeutic approach for individuals with ASD.

NF-κB is activated during the development and progression of autism [64]. Activated NF-κB promotes the synthesis and secretion of several inflammatory factors and disrupts cellular iron homeostasis. These factors, in turn, are involved in the feedback activation of NF-κB, thereby amplifying the inflammatory cascade reaction leading to irreversible neuronal damage that affects cognitive function and promotes anxiety–depressive behaviors [65]. Our data shows that PEPs inhibits p-NF-κB, thereby indicating its potential anti-neuroinflammatory activity. In neuroinflammatory diseases, a self-sustaining vicious cycle of neuroinflammation and neuronal apoptosis is observed [66]. Inflammatory mediators trigger the activation of the caspase cascade in the neurons, leading to cellular apoptosis via activation of the death receptors and the induction of mitochondrial dysfunction [67]. The apoptotic or necrotic neurons release damage-associated molecular patterns (DAMPs) such as HMGB1, ATP, and DNA fragments, which serve as “alarm signals” and activate the glial cells in the vicinity [68]. This perpetuates and escalates the inflammatory response and establishes a pathological cycle of protein aggregation, inflammation, and neuronal death. Activation of the NF-κB pathway begins with the recognition of external stimuli by cell surface receptors, with these signals ultimately converging on the IκB kinase (IKK) complex [69]. After activation, the complex specifically phosphorylates two key serine residues at the N-terminus of IκBα protein (Ser32 and Ser36 in humans) [70]. Subsequently, IκBα is catalyzed to undergo polyubiquitination modification, and ubiquitinated IκBα is rapidly degraded by the 26 S proteasome [71]. We found that PEPs can effectively rescue VPA-induced IκBα degradation, indicating that VPA acts through the IκBα/NF-κB pathway. The research observed that high concentrations of PEPs did not increase the expression levels of IκBα, possibly due to excessive activation of IKKβ leading to the degradation of IκBα [72]. Given the central role of the IκBα/NF-κB pathway in numerous diseases, it has become an attractive target for drug development. Enhancing the expression of IκBα or inhibiting its degradation may be an effective approach. Baicalein, a flavonoid compound, when administered during the subacute phase of brain ischemia–reperfusion injury, can suppress the NF-κB signaling pathway by reducing the phosphorylation of IκBα, thereby alleviating neuroinflammation and neuronal damage [73].

HSP90AA1, a highly conserved ATP-dependent molecular chaperone, plays a crucial role in maintaining cellular homeostasis by participating in the folding and stabilization of various signaling proteins, including AKT and NF-κB, which are widely involved in key biological processes such as signal transduction, cell cycle regulation, and apoptosis [74]. The downregulation of HSP90AA1 by PEPs may serve as an upstream event that affects the stability of these critical proteins, thereby synergistically inhibiting signaling pathways associated with hyperinflammation. In recent years, numerous network pharmacology studies have identified HSP90AA1 as a key target of various natural products and traditional Chinese medicine (TCM) formulations that have been used for the treatment of a range of diseases, including Alzheimer’s disease [75], metabolic syndrome [76], and myocardial ischemia–reperfusion injury [77]. Our study experimentally validated the effectiveness of PEPs in downregulating HSP90AA1 expression, thereby reinforcing the pivotal role of HSP90AA1 as a key therapeutic target. Our findings also demonstrate the anti-apoptotic and anti-inflammatory activities of PEPs.

However, this study has a few limitations. Firstly, the predictive outcomes of network pharmacology rely on the completeness and accuracy of existing public databases. This may introduce information bias. At the same time, focusing on monosaccharides as targets in network pharmacology research overlooks the impact of polysaccharide higher structures on biological activity. Secondly, the mouse hippocampal neuron HT22 cell line treated with VPA was employed as the in vitro model. Although prenatal exposure to VPA is a well-established environmental risk factor for ASD, it fails to replicate behavioral impairments associated with ASD in in vivo experiments. However, the HT22 cell line originates from the mouse hippocampus, whereas ASD involves functional impairments in a broad range of brain regions that cannot be fully recapitulated by in vitro models. Thirdly, PEPs have not been separated and purified into different components for further investigation. These limitations require us to separate the components and prove them through animal experiments in the future. However, these findings provide valuable insights for future research studies.

## 5. Conclusions

This study performed an integrated analysis of network pharmacology, molecular docking, and established an ASD cell model that can be used for screening biological activities. The research has identified the primary structure of PEPs and indicates that PEPs inhibit neuronal apoptosis and inflammation-related signaling pathways through modulation of the HSP90AA1, NF-κB/IκBα, and AKT/mTOR signaling pathways. The research findings deepen our understanding of the pharmacological activities of PEPs and open new avenues for the development of novel and safe treatment strategies for complex neurodevelopmental disorders such as ASD.

## Figures and Tables

**Table 1:** Ion chromatography conditions.

Parameters	Treatment Conditions
Column temperature	30 °C
Chromatographic column	Dionex^TM^ Carbopac^TM^ PA20 (3 × 150 mm)
Mobile phase	A: H_2_O, B: 15 mM NaOH, C: 15 mM NaOH and 100 mM NaOAc
Current velocity	0.3 mL/min
Injection volume	5 µL
Gradient elution degree	0~18 min (A:B:C = 98.8:1.2:0), 20~30 min (A:B:C = 50:50:0), 30.1~46 min (A:B:C = 0:0:100), 46.1~50 min (A:B:C = 0:100:0), 50.1~80 min (A:B:C = 98.8:1.2:0)
Detector	Electrochemical Detector

**Table 2:** The molecular weight contribution table of PEPs.

Peak Number	RT /min	Mp /kDa	Mw /kDa	Mn /kDa	PDI	Relative Peak Area %
1	11.822	1535.617	1580.196	1884.417	0.839	16.081
2	16.188	3.972	3.813	4.687	0.814	83.919

**Table 3:** Topological properties of the top 10 ASD-related PEPs targets.

Rank	Gene Symbol	Degree	Betweenness Centrality	Closeness Centrality	Neighborhood Connectivity
1	CASP3	60	0.6396	0.6866	33.8833
2	IL6	90	4.3283	0.5022	25.5667
3	HSP90AA1	85	3.3474	0.5737	27.2588
4	NF-κB1	60	1.3051	0.6196	33.8833
5	HSP90AB1	67	1.6469	0.5385	31.0597
6	AKT1	96	1.3130	0.6667	25.0625
7	STAT1	52	0.5135	0.5385	33.7500
8	mTOR	60	1.2658	0.5385	33.4667
9	HIF1A	66	1.6616	0.6667	29.9697
10	EP300	55	0.6798	0.5926	31.8364

## Data Availability

Raw data from the assays have been deposited in the Mendeley Data. Link to repository data: https://data.mendeley.com/preview/s4pknwrdtj?a=3761a082-2d6b-4f3e-97ec-219db30f5bd9 (accessed on 23 September 2025). Further inquiries can be directed to the corresponding author(s).

## Supplementary Materials

The following supporting information can be downloaded at: https://www.mdpi.com/article/10.3390/cimb47100796/s1.

## Abbreviations

The following abbreviations are used in this manuscript:

PEPs	*Enteromorpha prolifera* polysaccharide
ASD	autism spectrum disorder
VPA	valproic acid
