# Supplementary material for: Enteromorpha prolifera Polysaccharides Alleviate Valproic Acid-Induced Neuronal Apoptosis in a Cellular Model of Autism Spectrum Disorder
Source: Curr Issues Mol Biol. 2025 Sep 25;47(10):796. doi: 10.3390/cimb47100796 (PMC12563448; doi:10.3390/cimb47100796)
Supplement: Supplementary file 1 [file cimb-47-00796-s001.zip › cimb-3834842-supplementary.pdf]

Table S1. Box parameters of molecular docking box parameters

Targets	PDB ID	Coordinate center of the docking pocket space	The center size of docking pocket
AKT1	4EJN	center_x = 32.361	size_x = 51.39
		center_y = 44.735	size_y = 62.69
		center_z = 15.043	size_z = 64.75
CASP3	1RE1	center_x = 30.208	size_x = 59.85
		center_y = 102.225	size_y = 51.3
		center_z = 8.004	size_z = 50.35
HSP90AA1	3B25	center_x = 65.639	size_x = 44.25
		center_y = 15.353	size_y = 46.5
		center_z = 28.834	size_z = 45.75
HSP90AB1	7ULJ	center_x = -1.798	size_x = 98.35
		center_y = 101.513	size_y = 71.81
		center_z = -0.636	size_z = 98.35
MTOR	4HVB	center_x = 35.188	size_x = 97.65
		center_y = 3.695	size_y = 71.3
		center_z = 27.571	size_z = 75.95
IL6	1ALU	center_x = 2.599	size_x = 51.45
		center_y = -20.016	size_y = 51.45
		center_z = 8.749	size_z = 44.1
NF-κB1	1NFI	center_x = -5.372	size_x = 75.58
		center_y = 65.52	size_y = 85.52
		center_z = 45.296	size_z = 214.8
STAT1	7NUF	center_x = -96.375	size_x = 101.75
		center_y = -57.839	size_y = 74.0
		center_z = 228.094	size_z = 116.55
